# What google maps can do for biomedical data dissemination: examples and a design study

**DOI:** 10.1186/1756-0500-6-179

**Published:** 2013-05-04

**Authors:** Radu Jianu, David H Laidlaw

**Affiliations:** 1 School of Computing and Information Sciences, Florida International University, 11200 SW 8th St, Miami, FL 33199, USA; 2 Computer Science Department, Brown University, 115 Waterman St, Providence, RI 02912, USA

**Keywords:** Bioinformatics, Biological visualization, Data dissemination, Regulation networks, Design guidelines

## Abstract

**Background:**

Biologists often need to assess whether unfamiliar datasets warrant the time investment required for more detailed exploration. Basing such assessments on brief descriptions provided by data publishers is unwieldy for large datasets that contain insights dependent on specific scientific questions. Alternatively, using complex software systems for a preliminary analysis may be deemed as too time consuming in itself, especially for unfamiliar data types and formats. This may lead to wasted analysis time and discarding of potentially useful data.

**Results:**

We present an exploration of design opportunities that the Google Maps interface offers to biomedical data visualization. In particular, we focus on synergies between visualization techniques and Google Maps that facilitate the development of biological visualizations which have both low-overhead and sufficient expressivity to support the exploration of data at multiple scales. The methods we explore rely on displaying pre-rendered visualizations of biological data in browsers, with sparse yet powerful interactions, by using the Google Maps API. We structure our discussion around five visualizations: a gene co-regulation visualization, a heatmap viewer, a genome browser, a protein interaction network, and a planar visualization of white matter in the brain. Feedback from collaborative work with domain experts suggests that our Google Maps visualizations offer multiple, scale-dependent perspectives and can be particularly helpful for unfamiliar datasets due to their accessibility. We also find that users, particularly those less experienced with computer use, are attracted by the familiarity of the Google Maps API. Our five implementations introduce design elements that can benefit visualization developers.

**Conclusions:**

We describe a low-overhead approach that lets biologists access readily analyzed views of unfamiliar scientific datasets. We rely on pre-computed visualizations prepared by data experts, accompanied by sparse and intuitive interactions, and distributed via the familiar Google Maps framework. Our contributions are an evaluation demonstrating the validity and opportunities of this approach, a set of design guidelines benefiting those wanting to create such visualizations, and five concrete example visualizations.

## Background

Scientists today have access to many large datasets that describe biological processes. Advanced systems for visualizing such data exist but have associated costs that depend on a scientist’s computer abilities and familiarity with the data type and content. Thus, when handed unfamiliar datasets, researchers need to assess the time commitment these require and determine whether the analysis costs are justified. This scenario poses two limitations. First, researchers must judge a dataset’s relevance primarily by relying on textual descriptions and analyses provided by the data publisher. This method does not scale to large datasets where insights depend on a particular scientific question. Second, datasets deemed tangential to a user’s research may be discarded because of a low reward-effort ratio. These two limitations may lead to wasted analysis time and discarding of potentially useful data.

Raw datasets are commonly analyzed in one of the many stand-alone systems for biological data visualization developed over the past decade. These include software packages targeting microarray expression such as Clusterview [1], Hierarchical Clustering Explorer (HCE) [2], and Spotfire [3], systems for pathway and network analysis like Cytoscape [4], VisANT [5], Ingenuity [6] and Patika [7], or genome viewers such as Cinteny [8] and Mizbee [9]. Most such systems are aimed at complex data exploration and analysis and have associated overhead costs such as deploying, learning and operating the systems, data formatting, and adjusting parameters to create views.

Alternatively, large organizations and research groups sometimes choose to distribute data and analysis utilities as part of browsable web environments (e.g., tools on the NCBI website, web-based genome viewers). However, traditional web visualizations of biological data are restricted to small data volumes, limited visual encodings and keyhole analyses due to browser limitations [10]. Some developers overcame browser constraints by making their systems available as applets or to be run as client applications directly from websites [4,11]. However, in such approaches, users must still cope with overheads inherent to stand-alone applications such as adjusting visualization parameters, specifying data queries and learning features. Moreover, such websites are often difficult to setup and maintain, thus becoming prohibitively expensive for small data producers.

In this context we explore the benefits of using a tile-based approach to distribute raw data along with pre-rendered visualizations derived from it. Specifically, we explore the Google Maps API, a tile-based, pan-and-zoom interface that is well supported and highly familiar. As we will demonstrate in five examples, integrating our approach within new or established visualization systems would allow data producers (e.g., bioinformaticists, programmers assisting biologists in large labs) to create meaningful data views offline and easily distribute them online simply by copying a directory onto a webserver. Data consumers (e.g., individual researchers) could then readily access such data views in browsers. This removes the two limitations described in the previous paragraphs. First, it offers a simplified way of publishing data by eliminating the need for databases and complex client-server architectures. Second, it enables low-overhead access to readily analyzable views, thus facilitating lightweight analyses of datasets outside a researcher’s immediate focus. While perhaps not immediately suited for highly complex and on-the-fly analyses, we see this approach as particularly useful in augmenting traditional data publication.

Google Maps uses Ajax (asynchronous JavaScript and XML) technology to display images stored on a webserver in a user’s browser. This links our approach to calls for Ajax-based applications in biology [12,13] and a system implementation demonstrating how rendering can be performed on the server and resulting images served asynchronously to the browser [14]. However, the sole difference between this work and offline visualization systems is that control and display are done in a separate place from rendering and computation. Our research differs by attempting to reduce regular users’ effort in creating visualizations by assigning this task to experienced personnel, and by using an approach that rests on pre-rendered tiled visualizations frameworks such as the Google Maps API.

Google Maps or other pan and zoom frameworks have been recently used to display non-cartographic data. Closest to our work are X:MAP [15] and Genome Projector [16], which present implementations of genome browsers in Google Maps and CATMAID [17] which provides tiled imagery derived from microscopy and allows for annotation and collaborative work. Also similar is ZAME [18] which uses the zoom-and-pan paradigm to visualize graphs as adjacency matrices and looks similar to our heatmap representations. We apply the tile-based approach to a broader array of problems, by offering five concrete examples and providing evaluations of both Google-Maps-powered visualizations in general and of the specific visualization examples presented. It also differs from CATMAID by enabling the exploration of significantly larger data volumes.

Finally, from a theoretic and conceptual point of view, our work implements a range of aspects from the the Space-Scale diagram framework described by Furnas and Bederson [19], work which has inspired several results on multi-scale visualization systems [20], semantic zooming [21], and navigation paradigms for large zoomable spaces [22].

The work we present here was motivated and validated by collaborating on the Immgen project, a scientific effort aimed at generating a compendium of gene expression in immune cells. Our goal was to disseminate the project’s microarray data on the Immgen website. A collaborative design process revealed that the pre-rendered browser approach worked well here: data comes in large quantities, benefits from exploration, and requires hyperlinking to other data sources, biologists use well established visualizations, many of which 2D and requiring little interaction, and lab researchers are rarely eager to install and learn new applications. Finally, our collaborators were excited about replacing their database-driven, query-oriented website with something easier to maintain and more visually expressive.

The contributions of this paper lie in an evaluation across multiple visualizations of how Google Maps can help the biological domain, an exposition of design elements for building such visualizations, and a few algorithmic elements specific to each of our example viewers. Several of the elements featured in our work have been previously investigated by other authors but mostly in isolation. We also mention that we have described several of the visualization components featured in this article in other publications. Specifically, in [23] we describe the use of Google Maps to view genomic co-regulation data, in [24] we use Google Maps to increase the accessibility of visualizations of white matter structures in the brain, while in [25] we discuss how to display node-link diagrams of protein-protein interactions using a static map interface. These publications provide valuable details about the implementation of these visualizations and are featured as examples in this paper. Here we give a unified discussion of the use of Google Maps for visualizing biomedical data and provide an encompassing evaluation. As such, this is the first paper that approaches the use of Google Maps with an emphasis on evaluation and general design.

## Methods

This section introduces the Google Maps interface and five examples we have implemented using this technology. These examples are demonstrated in Additional file 1. Discussion of several design elements is deferred to the results section, which gives a more unified exposition on using Google Maps to display non-geographic data.

### Google Maps

We use the Google Maps API, an Ajax tile-based framework used to render large maps, to display our visualizations. It receives as input image data in the form of a set of small images, called tiles, that when assembled form the different zoom levels of the map. Each zoom level consists of a rectangular grid of tiles of size 2^*zoom*^×2^*zoom*^. The API decodes the zoom level and coordinates of the currently viewed map region to retrieve and display the visible tiles. The developer can load a custom set of tiles in the API by implementing a callback function that translates numerical tile coordinates and zoom level into unique paths to the custom tiles. The API provides basic functionally such as zooming and panning and allows programmatic extension or customization with markers and polyline overlays, information pop-ups and event management. The API can be easily integrated into any Javascript-powered web page.

### Gene co-expression map

**Figure 1:**
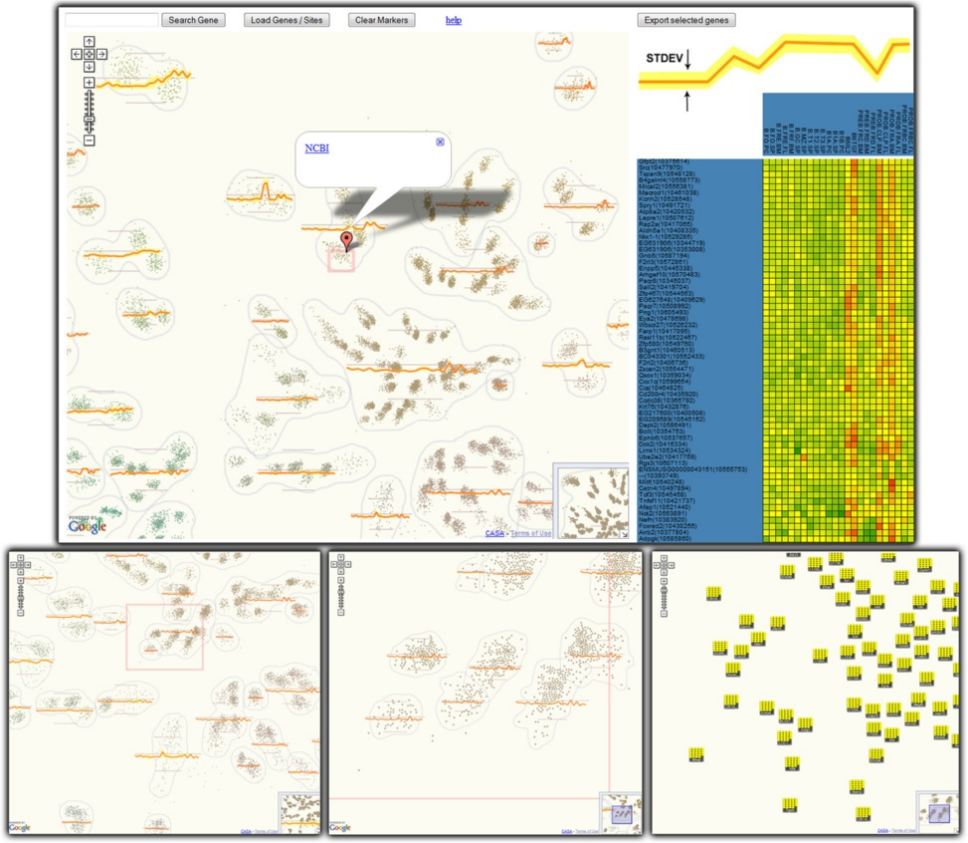
**Co-expression map of 23k genes over 24 cell types of the B-cell family.** The top view illustrates how maps are combined with client-side graphics: the map is at the center of the display while selecting genes on the map generates the heatmap on the right. Maps have multiple levels of zooming (bottom row), each with a potentially different representation. For example, genes are drawn as heatmap glyphs at the high zoom (lower right), and as dots at low zoom. Expression profiles of collocated genes are aggregated and displayed as yellow glyphs over the map. As zoom increases, expression profiles are computed for increasingly smaller regions. Interactions are not limited to zooming and panning; pop-up boxes link out to further data sources and selections of genes bring up a heat map (top panel).

In [23] we introduced a Google Maps browser viewer that displays co-regulation of large numbers of genes. Specifically, given genes with expression measurements over multiple cell types, we construct 2D projections that place genes so that their proximity is proportional to the similarity of their expression profiles (see Figure 1). In essence this is a dimensionality reduction problem similar to that proposed by Skupin [26,27].

We used a custom planar embedding algorithm inspired by HiPP [28] that introduces discrete cluster boundaries in the visualization (Figure 1). This addressed user feedback indicating that the lack of visible clusters detracts from analysis. In our implementation genes form groups based on their planar location. Such groups are enclosed by bounding curves and glyphs depicting the average-expression profile of each group are superimposed at the group’s location. The specificity of groupings is linked to zoom level (groups become smaller and tighter when zooming in). Similarly to [26] this was achieved by superimposing a hierarchical clustering and zoom-linked cut levels. As seen in Figure 1, we couple a standard Google Map implementation to client-side graphics (Protovis [29]) to display expression heatmaps of selected genes on demand. Users have the possibility to search for genes and highlight them via markers, and retrieve gene metadata in information pop-ups.

### Gene expression heatmaps

Given genes with multiple expression measurements over multiple cell types, we construct rectangular heatmaps. Each row corresponds to a gene and each column to a condition and each cell is a color-coded expression value. Rows and columns are arranged to place co-regulated genes and conditions together.

**Figure 2:**
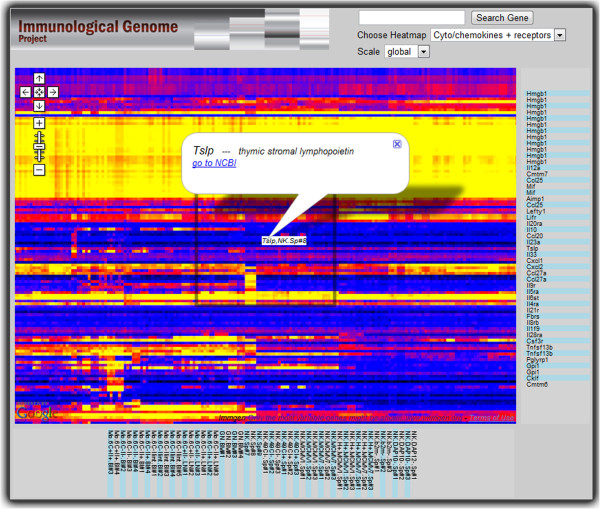
**Heatmap representation.** A heatmap representation is displayed as a map, with gene and cell-type axes implemented in Protovis attached at right and bottom. The axes are linked to the map’s zooming and panning so that users can identify which genes and cells they are looking at. Selection of an area of interest prompts the highlighting of the corresponding cell types and genes.

Figure 2 exemplifies a low-cost Google Map implementation using our collaborators’ color conventions. Protovis was used to attach at the right and the bottom of the map axes gene and condition labels. These are synchronized to the map’s zoom and pan so that labels for the currently viewed region of the heatmap remain within view. Mouse-over is used to display the gene-cell combination at a given heatmap cell while information pop-ups can be used to retrieve more detailed metadata. This representation is deployed and in use on the Immgen website.

### Genome browser

**Figure 3:**
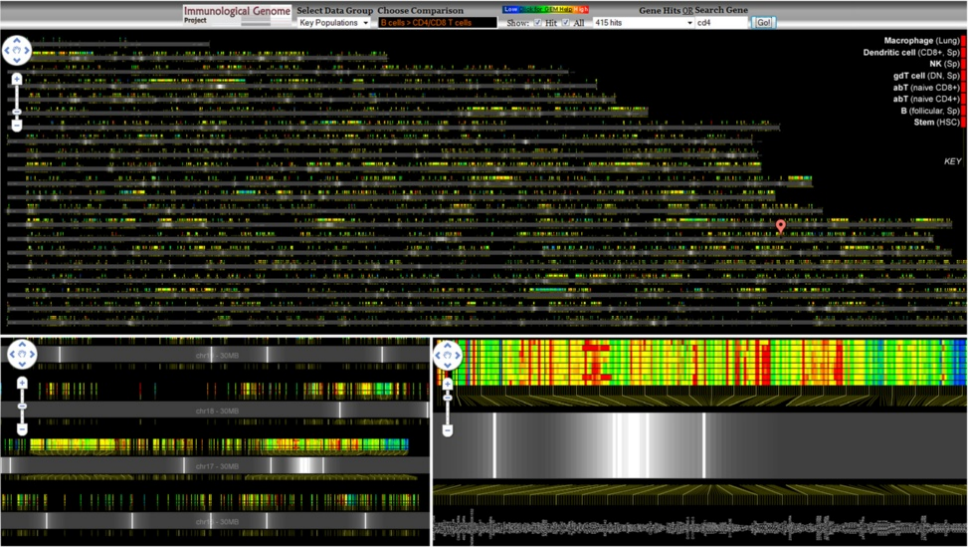
**Genome viewer as Google Map.** Gene expression data measurements over eight cell types of the entire mouse genome are mapped onto genome coordinates. The top view shows the general analysis framework as presented on the Immgen website; zoomed-in views appear at the bottom. Three types of visual queries can be performed, depending on the zoom. At an overview, lists of relevant genes can be highlighted using Google markers with custom icons - white lines with alpha gradients on each side - marking regions with interesting expression characteristics. At an intermediate zoom (lower left), regions with similar expression can be identified: a blue low-expression region is visible at center right. At a zoomed-in level individual expression values and gene names can be identified.

Given expression values over a set of conditions for any gene, we create color-coded expression glyphs at genes’ genomic coordinates (see Figure 3). Such representations can answer questions about correlations between gene function and genomic location.

Heatmap glyphs color-coding expression in multiple cells are created for each gene, using our collaborators’ color convention. A gene-name label is included for each gene. Chromosomes are arranged vertically, each extending horizontally. In response to user feedback, no space warping or distortions, such as in [9,30], have been used. The expression glyphs are mapped onto this space based on gene location. We use no aggregation of expression for different zoom levels because our collaborators felt that expression variability in co-located genes is sufficient to render aggregations meaningless.

Genes are not uniformly distributed on chromosomes; instead, regions with high and low gene density alternate. In high-density regions the space available to render a gene, assuming finite zooming, is limited and often insufficient to ensure visibility of the glyph elements. We therefore spread gene glyphs apart and anchor them with a leader line to their true genomic positions.

Gene search and highlighting of sets of genes are supported. The highlighting marker is an image with high alpha in the center and fading alpha towards the boundaries, so that the closer two highlighted genes are, the more their markers amplify each other. This ensures that regions with a high density of marked genes stand out in overview zooms (Figure 3).

### Protein interaction networks

In a fourth example we display protein interaction networks from online databases in browsers, using pre-rendered tiled visualizations. Such representations let proteomicists understand experimental data in the context of available information. The complete technical details of our implementation can be found in [25].

Network information is not intrinsically spatial, so that zooming and panning do not necessarily define useful data queries. Specifically, layout algorithms may place connected proteins far apart and zooming then splits them across multiple views. As described in [25] we use vertex splitting, a process which untangles graph layouts by duplicating nodes, to ensure that linked-proteins are co-located. Vertex splitting has been originally introduced by Eades and Mendonca [31] and revisited more recently by Henry *et al*[32] as node duplication. As a further design choice we use the city-versus-town distinction in a map analogy to filter out unimportant proteins at overview zoom levels. As in [33], this relevance measure is computed as a function of a protein’s intrinsic relevance and a relevance diffused from neighboring nodes.

**Figure 4:**
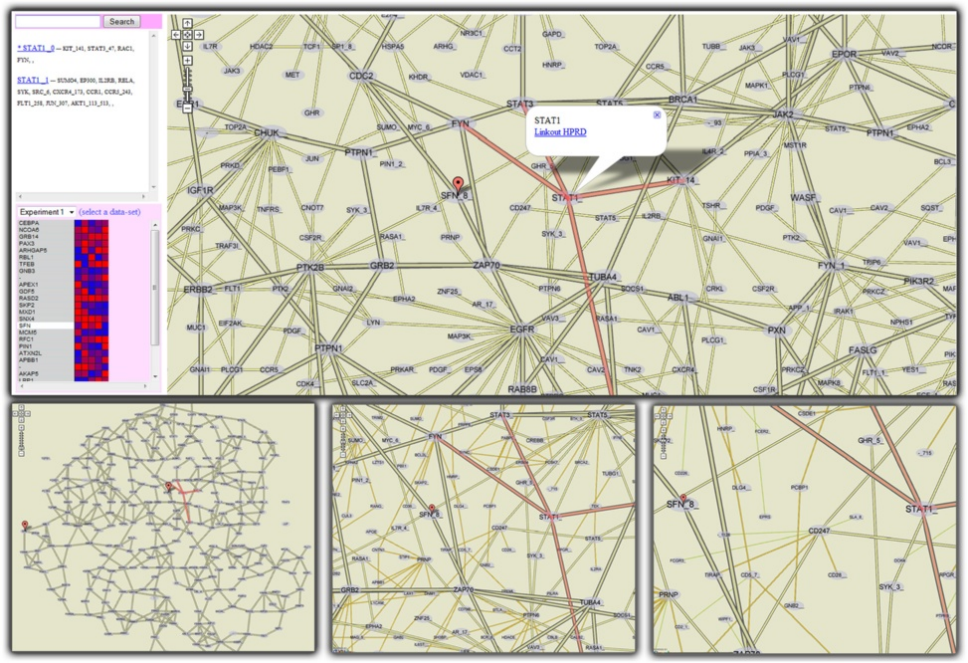
**Analysis of quantitative proteomic data in the context of a protein interaction network.** The top panel shows an overview of the analysis setup. Time-course proteomic data is displayed on the lower left. The experimental protein selected in the list is highlighted on the map. A second protein selected on the map has its interactors and meta-information displayed. All instances of this protein are listed on the upper left, together with their interactors. Three additional zoom levels are shown on the lower row; as zoom level increases, less relevant proteins are added to the display.

As shown in Figure 4, we use polyline overlays to show selected proteins, information pop-ups to display meta-data, and markers to highlight experimentally derived proteins. Vertex splitting generates multiple copies of the same protein. A window on the side of the map lists copies of selected proteins: clicking on list-items causes a jump to that copy’s location. Finally, proteomic experimental data can be loaded and displayed as heatmaps on the left-hand side of the map.

### Neural projections

In [34] we show how to construct planar representations of white-matter structures in the brain, starting from conventional 3D tractograms. Specifically, we cluster tracts using a curve-similarity measure, select centroid tracts for each cluster, and project these onto three principal projection planes: sagittal, coronal and transverse. These projections completely describe white matter in the brain and can be distributed as Google Maps. They enable scientists to navigate through sets of tractograms, analyze characteristics of major white-matter structures and find datasets exhibiting desired statistical properties.

**Figure 5:**
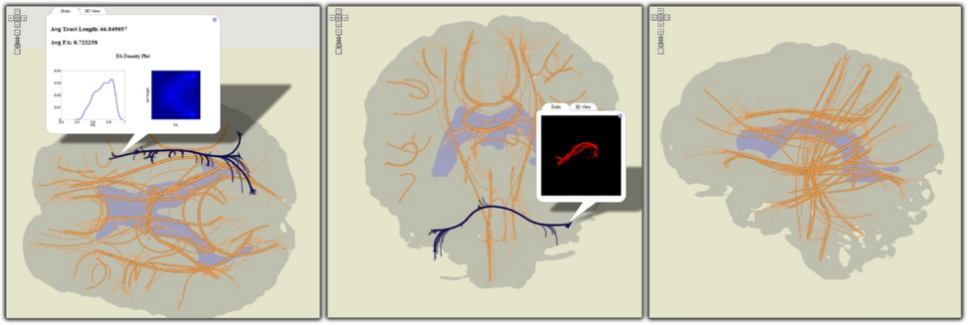
**DTI tractography data projected onto the transverse, coronal and sagittal planes.** Major tract bundles are represented schematically by their centroid tract; individual tracts in bundles are linked from the centroid bundle to their projected endpoints. Bundles can be selected and precomputed statistical data along with 3D poses of the tract bundle can be displayed.

As seen in Figure 5, tracts can be selected and highlighted on the projection map using polyline overlays. Selections rely on tract trajectory information that is exported along with the pre-rendered visualizations. Statistics in both textual and image form are pre-computed for each tract cluster, when the visualization is created, as are a few 3D poses as animated GIF images. This information can be retrieved in information pop-ups.

While this application domain is not tightly related to those of our other visualizations, its implementation furthers the design discussion that follows. Additionally, its evaluation brings to light several limitations of this approach that are perhaps peculiar to domains other than genomics or proteomics.

## Results

This section documents the main results of our work: a comprehensive evaluation demonstrating the benefits of using tile-based interfaces to disseminate biological data and a set of design guidelines for software designers who wish to build on this approach.

### Evaluation

An evaluation by domain experts of the viewers described in the previous section reveals strengths and limitations of the general approach as well as of each individual visualization example. We start with details about the evaluation procedure, then present feedback concerning the approach as a whole, and end with comments on each of the five visualizations.

#### Methodology

Twelve domain experts were involved in our evaluation. Four proteomic researchers from two separate labs evaluated the protein network. Five geneticists studying immune cells evaluated the co-regulation viewer, heatmap and genome browser. One of them, the Immgen coordinator, collaborated with us during the design and implementation of these three visualizations. Finally, three neuroscientists gave us input on the neural projections.

We showed the viewers to individual subjects and collected feedback. We first explained the visual encodings of the data and demonstrated the interactive features of the visualizations. We then let the subjects use the visualizations and encouraged them to give us their impressions during the process. Questions prepared in advance were also asked explicitly if our subjects didn’t touch on them during conversation. Open-ended questions were asked first, followed by more concrete inquiries on potential drawbacks or advantages.

#### Evaluation summary

This section presents feedback on the pre-rendered browser approach as a whole. 

**Ease of use:** Subjects rated ease of use higher than for other systems they were familiar with. They were excited to visualize data in browsers. The proteomicists in particular stated that this setup makes them more likely to use the visualizations, remarking that they prefer not to spend time installing software and learning new features.

**Users:** Potential users were identified as scientists lacking access to a computational infrastructure and those analyzing unfamiliar datasets. Our geneticists noted that new Immgen members spend considerable time becoming accustomed to data, and that such visualizations would support this process. One subject, contemplating her post-graduation life, removed from the bioinformatics support of Immgen, realized how helpful it would be if data was generally presented in this form.

**Use:** Unlike advanced analysis systems, we targeted exploratory, preliminary and casual browsing of data. Our subjects suggested using these visualizations to learn new datasets and for casual analysis while commuting or at home. Another suggestion was to use such applications to create small customized datasets from larger data volumes. Our subjects were most excited about the intuitiveness and low overhead and several noted that the interaction set was sufficient for their analysis tasks.

**Workflows:** The main workflow we identified was projecting familiar data elements onto existing data spaces. Geneticists would highlight their genes of interest into the co-regulation viewer; proteomicists would load experimental datasets and explore their interaction neighborhood in the interaction network.

**Interactivity:** Most subjects remarked that the implementations demonstrated were sufficiently complex for quick data analysis. Most were content with the feature sets, interaction and visualization provided, while some asked for more hyperlinking and metadata features.

**Drawbacks:** Perhaps unsurprisingly, it was the static nature of our approach that drew the most criticism. Even so, those expressing concern were in the minority: one geneticist and all three neuroscientists. The geneticist said the inability to customize what the visualization is showing would impede his analysis. He was, however, interested in disseminating his data in this form, indicating that he found the approach useful. This subject was a senior lab member highly familiar with the Immgen data used for the demonstration, which may explain his desire for flexibility. All three neuroscientists said that interactive fiber-tract selection mechanisms are indispensable in clinical white matter studies. Since selections in our visualization are restricted to pre-computed fiber clusters, they are insufficiently flexible. However, they noted that the approach is ideal for searching data repositories for candidate datasets for studies and for casually browsing data.

**Summary:** The most tangible feedback we received was the decision of the Immgen coordinator to switch the lab’s database-driven distribution system to our pre-rendered tile approach. He commented on the benefits of accompanying raw data with relevant visualizations. An important factor in his decision was the minimal overhead of both maintaining and using the systems.

#### Evaluation of individual viewers

Here we present feedback received for each of the five individual viewers. 

**Gene co-expression viewer:** Subjects found the co-expression projections useful in identifying how sets of genes of interest co-regulate in various cell combinations, and in finding other genes that exhibit expression patterns similar to known ones. One subject would also look for global patterns of co-regulation, possibly over multiple maps, and suggested we link maps in separate browser tabs.

The visualization was deemed intuitive and easy to use. Two users particularly liked the superposed expression profiles, stating that they summarized data well and could guide exploration. Users were also happy with the heatmap-upon-selection mechanism and with the ability to export selected sets of genes. Three out of five users were content with the pre-defined cell configurations imposed by the static visualization. The other two would have preferred to customize the cell types over which genes are projected, but noted that in their domain only a few cell subsets were biologically meaningful (e.g. corresponding to cell families or lineages).

**Gene expression heatmap:** Our collaborators often publish static heatmap images as large as 2000 rows by 500 columns. The absence of any interaction, however, is an important limiting factor, which is what motivated our implementation. Only two of our five evaluation subjects used heatmaps at this scale in their analysis and were able to provide feedback. They were excited about our visualization and noted that the mouse-over functionality, information pop-ups, and sticky axes were sufficient for their analytic needs. A single extension was recommended: zooming along a single dimension (genes or cells) to create a visual aggregation effect that could answer some scientific questions. Our collaborators adopted the interactive heatmaps and made them operational on the Immgen website.

**Genome browser:** Initial feedback identified the need for an overview analysis of gene expression in the genome space, in particular the extent to which adjacent genes share expression patterns.

The viewer does not employ semantic zooming such as aggregating expression values over contiguous genomic regions. Instead we relied on additive visual cues of individual items that create salient expression patterns when zoomed out. Our collaborator suggested this design to avoid erroneous aggregation effects and subsequent feedback suggested that it was indeed effective. The ability to highlight genes identified by specific queries (Figure 3) was also considered useful. Using it, our subjects observed that genes with comparable patterns of activity tend to be dispersed and that co-regulated clusters exist but are relatively rare, contrary to their prior beliefs. This feedback was provided by two subjects interested in overview analyses of regulation patterns. Our other three subjects were less interested in genomic mappings were unable to comment on the usefulness of this particular visualization.

**Protein interaction networks:** Our proteomicists were excited about looking at interaction networks in their browsers. The consensus was that the setup is highly effective and that they would choose it over other systems they were familiar with. The interactivity of the system was judged appropriate, with more metadata the feature most commonly requested.

Our subjects’ unanimous opinion was that relevance filtering was intuitive. They noted that it corresponded to how they normally approach a new network: identify important or familiar proteins and then drill down to learn more about their neighbors. Another comment was that seeing familiar proteins and connections early reinforces their confidence in the visualization. All subjects thought that the heuristics used to compute the relevance of proteins were appropriate. Three subjects stated that multiple copies of proteins resulting from vertex splitting would not obstruct their analysis. One even said he liked the approach because it made proteins’ interaction neighborhoods more apparent. The fourth subject said that protein duplicates are undesirable but acceptable as long as they can be easily explored. He noted that the copies-list on the left (Figure 4) lets him to do this efficiently.

**Neural projections:** The neuroscientists we interviewed commented that quantitative clinical studies on white-matter tractograms require precise bundle selections, thus rendering interactivity indispensable. However, they pointed out the unique opportunities offered by our visualizations: collaborating with other scientists by sending links, being able to look at datasets anywhere, any time, and browsing through datasets before importing a model into a desktop application. The evaluation led us to conclude that static maps are less suited for the 3D domain where complex interactions are needed, but can occupy a task-specific niche such as collaborative work and casual analysis.

### Design

Here we describe how to leverage the features of the Google Maps API in the context of data visualization. The design elements we present are a distillation of the feedback presented in the previous section and of the design and development that produced the visualizations featured here.

#### Overview

**Data size and specification:** To compensate for their static nature, pre-rendered visualizations should en-compass all data associated with a scientific problem. Thus, a visualization can be useful for many queries, since data specification can be done during visualization through zooming, panning and highlighting. Individual visualizations sometimes need to be adapted to suit this approach. Our protein interaction networks use vertex splitting to enable queries by zoom-and-pan and a zoom-linked filter to address clutter. Our co-regulation map uses expression glyphs that guide users towards gene groups with specific expression patterns.

**Use:** Unlike advanced analysis systems, we have only targeted exploratory, preliminary and casual browsing of data or lightweight analysis tasks. It is thus hard to determine how suited this approach is in the context of more complex functionality.

**Users:** Users can be divided into data consumers and data producers. In our experience, the former often perceive a dataset to have a low reward-effort ratio because they are unfamiliar with the type of data, are generally computer averse or lack access to a computational infrastructure. The browser visualizations targeting such users should be sparse and intuitive. This may seem self-evident, but state-of-the-art visualization systems commonly require scientists to understand visualization-specific jargon (e.g., select a specific graph-drawing algorithm). Data producers want to distribute visualizations along with their raw data so that fellow researchers need not run their own analysis. Data producers will use an interactive system to create the browser visualizations. The assumption is that they are specialists in the data they are distributing, so that a system can use more complex visualization metaphors.

**Development overhead:** Development overhead can vary greatly among visualizations: our heatmaps are just static images augmented with basic interactivity, co-regulation information had to be first projected in 2D, and protein interaction networks required an entirely new drawing algorithm. A simple heuristic is that the overhead depends on the effort required to planarize the information displayed (e.g., relational data is harder than projected multidimensional data) and on the amount of data shown.

**Deployment:** Google Maps visualizations can be designed to work without dependencies on databases and server-side scripting. In such cases they can be deployed by simply copying a directory structure to a web server. This was an important factor for our collaborators in deciding to adopt this mode of representation.

#### Interactivity

While reiterating that complex interactions are not the focus of this approach, we give below a few interaction patterns common in visualization that are possible in implementations based on Google Maps. 

**Selection/Brushing:** For selection, positions of selectable elements have to be exported in data files, along with the pre-rendered visualization. This information is used to translate coordinates of mouse events into selections. In the co-regulation viewer and heatmap, users select genes by drawing enclosing rectangles. In the white-matter visualization we export curve trajectories for each tract-cluster, and use the proximity of a mouse click to a curve as a selection heuristic.

**Highlighting:** Elements selected through interaction or search can be highlighted using markers or polylines (traditionally used to highlight routes in digital geographic maps). Figure 1 illustrates a group of selected genes identified by markers. Polylines are used to implement Munzner’s constellation technique [35] on the protein interaction network (see Figure 4) and highlight tract-cluster trajectories on the white-matter visualization. Finally, images shown as markers can be customized to create more complex effects. In the genome browser for instance, multiple co-located markers with alpha gradients create an additive visual effect.

**Semantic zooming:** Our protein interaction network illustrates semantic zooming by displaying additional proteins with each increase in zoom level. The map framework allows developers to show different images at each zoom level. A scene can thus be pre-rendered at different zoom levels, each with its own visual abstractions. Two important factors to consider are that a visualization can have only as many abstractions as zoom levels and that exported images double in pixel size with each additional zoom level. This should be taken into consideration in designing the number of abstractions, as thirteen-level visualizations are infeasible to distribute (see next section).

**Filtering:** Semantic zooming can be used to implement filtering. As mentioned before, our protein interaction network (Figure 4) illustrates this concept. While not implemented in any of our visualizations filtering could also be achieved by rendering multiple complete tile-hierarchies for pre-determined filtering conditions. Completely dynamic filtering is infeasible using pre-rendered visualizations.

**Data aggregation/abstraction:** In our co-regulation viewer we average expression values over groups of genes at varying levels of specificity. In the genome viewer we contemplated displaying aggregated expression values over larger genome regions at overview zooms to deal with gene density, but chose a different approach following user feedback. Semantic zooming is, however, a good way to implement varying degrees of data abstraction. Another way is to use combinations of markers with custom icons to create glyphs that show aggregated data; this has the advantage that such effects can be created programmatically at run time. A simple example is seen in our genome browser where selection glyphs create an aggregated visual effect.

**Details on demand:** Figures 1, 2, 4 and 5 illustrate how information popups are used to retrieve information about visualization elements. Figure 5 shows how pre-computed statistical data and 3D-poses can even offer different perspectives of selected data subsets. A second detail-on-demand implementation is shown in Figure 2: mouse hovering generates a tooltip overlay. For more interactivity, browser-side graphics can be coupled with Google Maps. The co-regulation map (Figure 1) uses Protovis to show expression values of user-selected genes as heatmaps. We note that information used in the detail views (e.g. expression values, 3D-poses etc) must be exported along with the rendered tiles.

**Overview+Detail:** The implicit Overview+Detail mechanism in Google Maps is the mini-map. However, more complex interactions can be achieved with browser-side graphics or multiple synchronized Google Maps on the same page. The closest feature to this in our implementations is the dynamically generated heatmaps in the co-regulation viewer. However, it would be easy to extend the protein interaction network by a linked Protovis viewer that displays local network information for selected proteins.

**Figure 6:**
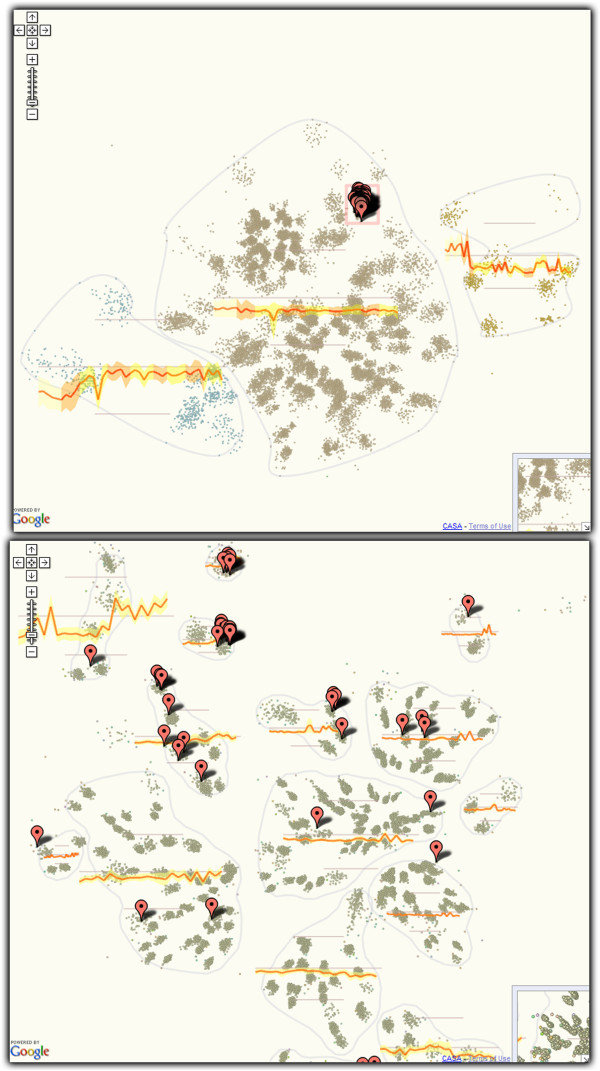
**Linked co-regulation maps of the T-cell (top) and B-cell (bottom) families.** A selection in the T-cell viewer is reflected in the B-cell viewer. A few groups of genes that are co-regulated in both cell families appear in the upper part of the B-cell map.

**Brushing and Linking:** Two of our evaluation subjects noted that linking several of our visualizations together can be beneficial. For example, linking co-expression views (e.g. for different cell families) can answer questions about conservation of gene function over multiple conditions. This functionality was implemented for the co-expression maps using browser cookie-polling, as shown in Figure 6. We also hypothesize that such brushing and linking functionality could be used to link data maps, external data sources, and other analytic web services together to create more complex environments.

#### Improving performance

Below are a few considerations for improving the performance of tiled visualizations.

None of our visualizations required more than nine zoom levels. Assuming a tile size of 256 pixels, these translate into square images with 2^8^∗256=65536 pixels on the side, at the largest zoom level. Furthermore, the number of tiles quadruples at each additional zoom level such that these visualizations consisted of $\overset{8}{\underset{i=0}{\sum }}2^{i}*2^{i}=87381$ image files. Efficient image compression is desirable to reduce space requirements and speed up tile loading. Tile numbers can also be reduced by exploiting that visualizations often contain areas of empty background. Thus, many tiles can be represented by a single background-tile. Coordinates of background tiles are exported at the time of rendering and subsequently decoded by the Javascript implementation. Empty tiles are usually compressed into smaller files by default (due to uniform coloring) and their number is visualization dependent. Still, performance gains remain meaningful and typically grow considerably with increases in a visualization’s zoom levels. Table 1 summarizes these improvements on several of our visualizations.

**Table 1:** Number of tiles and disk space analysis

	**All tiles**		**Non-empty tiles**	
	**PNG**	**JPG**	**PNG**	**JPG**
Co-reg.	(5461 37.6)	(5461 39.9)	(3505 35.1)	(3505 30.2)
Heatmap	(5461 23)	(5461 29.4)	(2811 12.7)	(2811 19)
Networks	(5461 32.2)	(5461 33.6)	(4620 29.8)	(4620 25.3)
Brain	(5461 37.6)	(5461 39.9)	(3505 34.1)	(3505 32.2)
Genome	(5461 35.1)	(5461 38)	(4051 27.1)	(4051 30.3)
Genome*	(87381 263.4)	(x x)	(17630 100)	(x x)

Number of tiles and disk space(MB) for the five visualizations with different image compression (PNG vs. JPG) and all tiles vs. non-empty tiles. First five rows stand for visualizations with 7 zoom levels; the last row corresponds to a 9 level genome browser.

As mentioned in the previous section, interaction and data on demand rely on exporting additional information at rendering time that must be fetched and used by the browser visualization. Loading this data at once, during initialization, can freeze the visualization and result in large memory loads. Instead, in line with the tile approach, the information should be split in multiple files and retrieved only when an interaction demands it. For example, information about the shape of the curves in the white-matter visualization is split over a 10×10 grid spanning the visualization. Upon a mouse click, the corresponding cell content is fetched and tested for intersections. If an intersection with a tract cluster is found, a file containing information about this cluster (e.g., cluster trajectories for highlighting, metadata to be displayed in information pop-ups) is retrieved. This ensures that visualizations remain responsive during interactive tasks.

## Discussion

There are several differences between traditional visualizations and the tile-based design we explored in this paper. First, instead of the data-query-specification/recomputed-visualization paradigm, our examples contained most data associated with a biological problem, and querying was essentially done through zooming and panning. Second, while traditionally end users are responsible for constructing visualizations, our evaluation suggests that in some cases placing the construction of visualizations in the hands of bioinformatics staff in larger labs, such that they are computed only once and become readily available for users to analyze, can be useful in several scenarios. Finally, we showed that fast and intuitive access to visual perspectives of a dataset, even if less flexible then complex systems in terms of interaction and queries, can help in some cases accelerate analysis.

As suggested by our evaluation, the low-overhead tile based approach we exemplify seems to be particularly attractive to researchers lacking access to a strong computational infrastructure, for unfamiliar datasets, and for casual data browsing. Our evaluation of the white matter visualization shows that in other domains this approach might be more narrowly useful. From our experience, the Google Maps API can also be a useful medium for gathering feedback on visual encodings, possibly developed as part of another system. Collaborators are more likely to provide feedback on visualizations that they can access and use with minimal overhead than on ones they must install and learn. Furthermore, concerns such as deployment and platform, rendering speed and interactivity, GUI and data formats become non-issues.

This work explores only the Google Maps API. However, we hypothesize that other Ajax tiled approaches would probably also be suitable for this approach. More generally, zoom-and-pan frameworks (e.g. Bing Maps API, Silverlight, OpenZoom) can be used in conjunction with a subset of the design elements discussed in this paper to develop similar visualization. Moreover, the development of a tiled frame-work designed to support data visualization rather than geographical maps could prove useful. Such a framework, if open source, would also alleviate concerns about licensing, support and stability associated with commercial products. Principles of sparsity and intuitiveness should remain the foundation of tile frameworks, since the proposed browser visualizations should not seek to rival complex systems.

**Figure 7:**
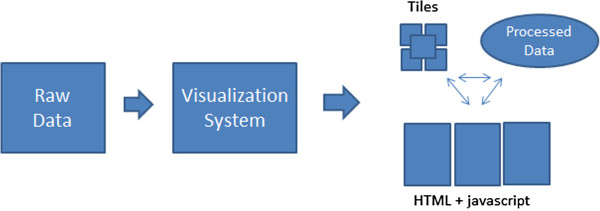
**Creating GoogleMap visualizations.** Raw data is entered into a stand-alone visualization system which outputs Google Maps as an image tile pyramid, a set of javascript webpages, and a set of data files to support Google Maps interactivity.

We have tested our approach by extending an existing visualization environment with Google Maps capabilities. This process involved adapting existing viewers to the particularities of Google Maps using the design guidelines described in this paper and extending rendering such that it could be performed offline, on tiles, rather than just on the screen. This process is shown in Figure 7. We note that any visualization system or visualization framework (e.g. Cytoscape [4], Prefuse [36]) could be augmented with the capabilities of outputing Google Maps rendering. Our particular system has not yet been released but our results suggest that scientists would benefit if more established visualization systems, such as the ones mentioned, would incorporate methods of exporting user created views as GoogleMaps. As a future direction we envision a web-service, extensible by modules, that would not only allow data producers to upload readily created data maps, but also enable individual researchers to upload their data and have visualizations created and published on the fly.

Users in our evaluation were excited about the collaboration facilities offered by maps. Exchanging interactive images rather than static ones and sending links rather than datasets was positively received. Pre-rendered visualizations are well suited for collaborative work, since they ensure that each user has the same view of the data and that shared comments target the same visualization elements. We would like to add annotation capabilities to our maps to let researchers exchange ideas.

Finally, an important component of visualization research is understanding how visualizations are used. Due to the minimal interaction advocated, maps should be easy to instrument. In fact, our deployed maps have been instrumented using the Google Analytics framework.

## Conclusions

A series of cognitive studies led Hegarty *et al*[37] to conclude that “cognitive science research indicates that the most effective visual representations are often sparse and simple. When given control over interactive visualizations, people do not always use these technologies effectively or choose the most effective external representations for the task at hand.” We presented a low-overhead approach that can facilitate browsing for a range of unfamiliar scientific datasets, that relies on pre-computed visualizations carefully prepared by data experts for distribution with sparse interactions, so that end users can access readily analyzed views of scientific data. We build on the familiarity of the Google Maps framework and leverage its functionality to distribute those views. Our primary contributions are an evaluation demonstrating the validity and opportunities of this approach and a set of design guidelines benefiting those wanting to create such visualizations. Additional contributions include five concrete example visualizations.

## Availability of supporting data

Much of the data used in this paper is published as part of the Immgen project at http://www.immgen.org. Protein interaction data has been extracted from the Human Protein Reference Database (HPRD) at http://wwww.hprd.org.

## Competing interests

Both authors declare that they have no competing interests.

## Authors’ contributions

RJ designed, implemented, and evaluated the visualizations presented in this paper. DL participated in the design of the visualizations, coordinated the collaborations, and helped to draft the manuscript. Both authors read and approved the final manuscript.

## Supplementary Material

Additional file 1 A web-based demonstration of four Google Map visualizations: gene co-expression maps, a heatmap representation, a genomic mapping, and a protein interaction network. Click here for file
