# Supplementary material for: What google maps can do for biomedical data dissemination: examples and a design study
Source: BMC Res Notes. 2013 May 4;6:179. doi: 10.1186/1756-0500-6-179 (PMC3658873; doi:10.1186/1756-0500-6-179)
Supplement: Additional file 1 — A web-based demonstration of four Google Map visualizations: gene co-expression maps, a heatmap representation, a genomic mapping, and a protein interaction network. [file 1756-0500-6-179-S1.htm]

Below are links to map visualization demos


|  |  |
| --- | --- |
| Below are links to map visualization demos. These implementations have been tested on Firefox and Chrome and are known to not work on Internet Explorer due to Protovis compatibility issues. |  |
| Gene co-expression in the BCell family |  |
| Gene co-expression in the TCell family |  |
| Heatmap represention of gene expression |  |
| Genomic mapping of gene expression in BCells |  |
| Protein network |  |
|  |  |
